# Small Incision Lenticule Extraction (SMILE) for Moderate and High Myopia: Seven-Year Outcomes of Refraction, Corneal Tomography, and Wavefront Aberrations

**DOI:** 10.1155/2020/3825864

**Published:** 2020-04-22

**Authors:** Fei Xia, Yang Shen, Tian Han, Jing Zhao, Haipeng Xu, Xingtao Zhou

**Affiliations:** ^1^Department of Ophthalmology and Optometry, Eye and ENT Hospital, Fudan University, Shanghai, China; ^2^NHC Key Laboratory of Myopia (Fudan University), Laboratory of Myopia, Chinese Academy of Medical Sciences, Shanghai, China; ^3^Shanghai Research Center of Ophthalmology and Optometry, Shanghai, China

## Abstract

**Purpose:**

To investigate the long-term outcomes of refraction, corneal tomography, and wavefront aberrations after small incision lenticule extraction (SMILE) for moderate and high myopia.

**Methods:**

Prospective, nonconsecutive case series. A total of 26 patients (26 eyes) who underwent SMILE from May 2010 to March 2013 at the Fudan University Eye and ENT Hospital (Shanghai, China) were enrolled. The periods of follow-up were 1 month, 1 year, 5 years, and 7 years after surgery. The routine eye examinations included uncorrected distance visual acuity (UDVA) and corrected distance visual acuity (CDVA), manifest refraction, and corneal tomography.

**Results:**

All surgeries were executed without any complications. At the final visit, an UDVA of 20/20 or better was achieved in 26 eyes (100%) and 11 eyes (42%) exhibited no change in CDVA. 9 eyes (35%) gained one line, 6 eyes (23%) gained two lines, and no eyes lost CDVA. 24 eyes (92%) and 26 eyes (100%) were within ±0.5 D and ±1.00 D of the target refraction, respectively. A mean refractive regression of −0.17 D was observed between 1 month and 7 years postoperatively. Mean corneal front curvature (MCFC) was significantly decreased between pre- and post-SMILE surgery (*P* < 0.0001). Higher-order aberrations (HOAs) and vertical coma were significantly increased after SMILE compared to those measured before surgery (all *P* < 0.001). There were no significant differences in trefoil and spherical aberration between pre- and post-SMILE surgery (all *P* > 0.05).

**Conclusion:**

SMILE is an effective, safe, and stable procedure for moderate and high myopia, with relatively constant corneal stability and wavefront aberrations. This trial is registered with ChiCTR-ONRC-13003114.

## 1. Introduction

As opposed to flap-based surgical procedures, small incision lenticule extraction (SMILE) is a minimally invasive refractive surgical technique that involves the manual removal of femtosecond laser-created intrastromal lenticule through a small side cut. Sekundo et al. [1] first introduced this new procedure in 2011. The first clinical study [2] of femtosecond lenticule extraction in China was also reported in 2011. Numerous clinical studies [3–9] suggested that SMILE is a safe and effective procedure for myopia correction, and the follow-up periods in these studies mostly spanned from 3 months to 3 years, but rarely up until 4 years [10–14]. In 2019, we [15] have previously compared the 5-year outcomes of SMILE and femtosecond laser-assisted LASIK (FS-LASIK) in patients with myopia. To date, more than 2 million SMILE procedures have been performed worldwide. Its long-term safety and efficacy have received enormous attention.

It is noted that the long-term observation of corneal stability and wavefront aberrations following SMILE is of great significance. The present study aims to evaluate the seven-year outcomes of refraction, corneal tomography, and wavefront aberrations after SMILE for moderate and high myopia.

## 2. Materials and Methods

### 2.1. Subjects

A total of 26 patients (26 eyes) who underwent SMILE between May 2010 to March 2013 at Fudan University Eye and ENT Hospital (Shanghai, China) were recruited in this prospective study, and only the right eye was included in this study. The mean follow-up period was 82.53 ± 8.5 months (range 73 to 101).

The inclusion criteria were as follows: (i) dissatisfaction of wearing glasses or contact lens; (ii) corrected distance visual acuity (CDVA) of 20/25 or greater; (iii) age 18 years or older; (iv) manifest refraction spherical equivalent (MRSE) of −1.0 to −9.0 D and manifest cylinder of 0 to −6.0 D; (v) a stable refraction state for at least two years; (vi) residual corneal stromal bed thickness of more than 280 *μ*m; and (vii) no use of contact lenses over the last 2 weeks. Patients with suspicious keratoconus, severe dry eyes, cataract, retinal detachment, or other ocular disorders were excluded. In addition, those with systemic diseases such as diabetes and connective tissue diseases were excluded as well. The demographic and clinical characteristics of patients with moderate and high myopia are shown in Table 1.

This study adhered to the tenets of the Declaration of Helsinki and was approved by the Medical Ethics Committee of Fudan University Eye and ENT Hospital, Shanghai, China. Written informed consent was obtained from all participants before enrollment.

### 2.2. Surgical Procedure

The surgical technique has been detailedly described by Miao et al. [16]. VisuMax femtosecond laser system (Carl Zeiss Meditec AG, Jena, Germany) was set to a pulse energy of 130 nJ and repetition rate of 500 kHz. Additional parameter settings were as follows: 6.5 to 6.8 mm lenticule diameter, 100 to 110 *μ*m cap thickness, 7.5 to 7.8 cap diameter (larger than 1 mm of lenticule), and a 4.5 mm side cut (90 degrees) at 12:00 clock. All procedures were performed by the same experienced surgeon (XZ).

Postoperative medications included levofloxacin 4 times a day for 1 week and sodium hyaluronate 4 times a day for 1 month. Meanwhile, 0.1% fluorometholone solution was tapered gradually once every three days from 8 to 1 times daily.

### 2.3. Main Outcome Measures

The following parameters were assessed preoperatively at 1 month, 1 year, 5 years, and 7 years postoperatively: (i) uncorrected distance visual acuity (UDVA) and CDVA; (ii) manifest and objective refraction; (iii) intraocular pressure (Tonoref II, Nidek, Japan); (iv) slit-lamp examination; and (v) central corneal thickness (CCT), mean corneal front curvature (MCFC), mean corneal back curvature (MCBC), posterior central elevation (PCE), preoperative PCE subtracted from postoperative PCE (ΔPCE), and wavefront aberrations.

CCT, MCFC, MCBC, PCE, ΔPCE, and wavefront aberrations were measured using a corneal tomography (Pentacam, Oculus GmbH, Wetzlar, Germany). MCFC, MCBC, and PCE were delineated in the central 4 mm area above the 8 mm reference best-fit sphere. ΔPCE was defined as the difference between preoperative PCE and postoperative PCE at two time points.

Corneal wavefront aberrations were evaluated at a 5 mm analysis diameter. The coefficients of spherical aberration (*z*_4_^0^), the vertical coma (*z*_3_^−1^), the horizontal coma (*z*_3_^1^), the vertical trefoil (*z*_3_^−3^), horizontal trefoil (*z*_3_^3^), and the root mean square (RMS) of total higher-order aberrations (HOAs) up to fourth order were calculated using Zernike polynomials.

### 2.4. Statistical Analysis

All data were analyzed using SPSS version 23.0 statistical software (SPSS Inc., Chicago, IL, USA). Continuous variables were presented as mean ± standard deviation (SD), while categorical variables were shown as frequency or percentage. Pearson correlation coefficient was used to determine the correlation between the attempted and achieved spherical equivalent. Repeated measure analysis of variance was performed to assess the standardized differences of preoperative and postoperative visits at various time points. The post hoc test with Bonferroni correction was used to compare the data between two different time points. *P* values of less than 0.05 were considered statistically significant.

## 3. Results

All operations were completed smoothly without any intraoperative and postoperative complications. No case of corneal ectasia was found in the present study.

### 3.1. Efficacy and Safety

**Figure 1:**
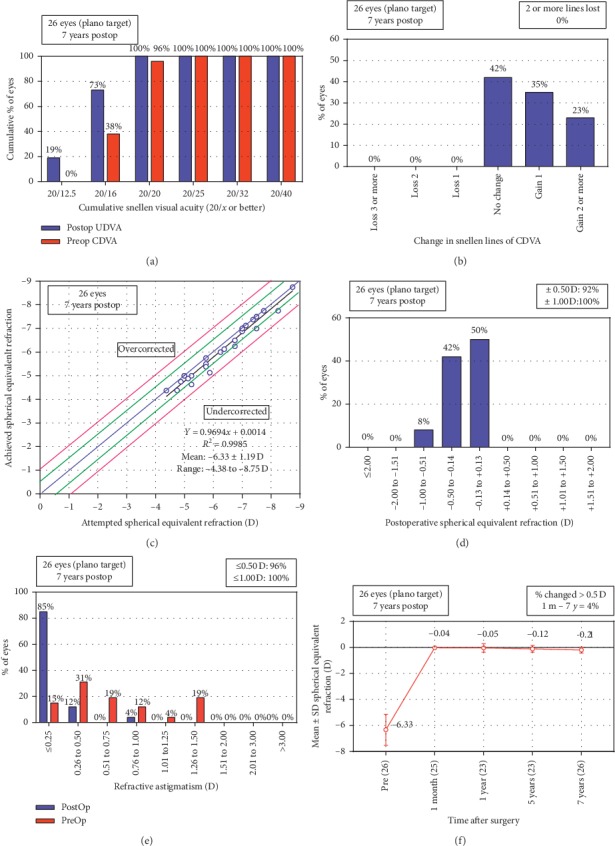
Refractive outcomes at 7 years postoperatively for 26 eyes for moderate and high myopia (UDVA = uncorrected distance visual acuity, CDVA = corrected distance visual acuity, D = diopters, Preop = preoperative, and Postop = postoperative). Uncorrected distance visual acuity (a); change in corrected distance visual acuity (b); spherical equivalent attempted vs achieved (c); spherical equivalent refractive accuracy (d); refractive astigmatism (e); stability of spherical equivalent refraction (f).

The efficacy index (postoperative UDVA/preoperative CDVA) was 1.14 ± 0.19 (Figure 1(a)). Among the 26 eyes, all eyes (100%) achieved an UDVA of 20/20 or better, 19 eyes (73%) achieved an UDVA of 20/16 or better, and 5 eyes (19%) achieved an UDVA of 20/12.5 or better.

The safety index (postoperative CDVA/preoperative CDVA) was 1.19 ± 0.20 (Figure 1(b)). Eleven out of 26 eyes (42%) demonstrated no change in CDVA. 9 eyes (35%) gained one line, 6 eyes (23%) gained two lines, and no eyes lost CDVA.

### 3.2. Predictability and Stability

As shown in Figure 1(d), 24 eyes (92%) and 26 eyes (100%) were within ±0.5 D and ±1.00 D of the targeted refraction, respectively. Meanwhile, 25 eyes (96%) and 26 eyes (100%) were within ±0.5 D and ±1.00 D of refractive astigmatism, respectively (Figure 1(e)). A scatter plot of the attempted versus the achieved correction (spherical equivalent) is illustrated in Figure 1(c).

The mean values of MRSE went from 0.04 ± 0.15 D at 1 month postoperatively to −0.21 ± 0.23 D at 7 years postoperatively (*P*=0.023). A mean refractive regression of −0.17 D was observed between 1 month and 7 years postoperatively, which corresponded to a regression rate of −0.02 D per year. In addition, the change in spherical equivalent refractive error was more than 0.5 D in 1 (4%) out of 25 eyes (Figure 1(f)).

### 3.3. Corneal Stability Analysis

**Figure 2:**
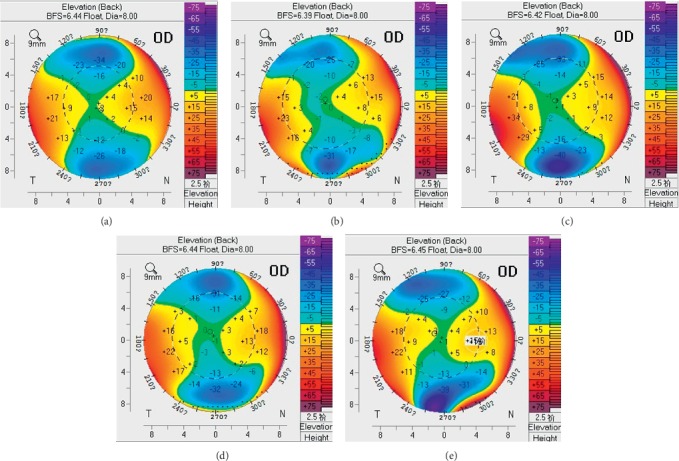
Comparison of posterior corneal surface maps obtained by corneal topography preoperatively (a), 1 month (b), 1 year (c), 5 years (d), and 7 years (e) after small incision lenticule extraction (SMILE).

The outcomes of corneal stability analysis are presented in Table 2. The mean values of CCT and MCFC at 7 years after SMILE were significantly decreased compared to those measured preoperatively (all *P* < 0.001). However, PCE and MCBC showed no significant change between before and 7 years after surgery (*P*=0.180), and no significant differences were found in the values of ΔPCE at different time points after surgery (*P*=0.463).  Figure 2 shows the differences between the posterior corneal surface maps obtained by corneal tomography preoperatively (a) and 1 month (b), 1 year (c), 5 years (d), and 7 years (e) after SMILE.

### 3.4. Corneal Wavefront Aberrations

Table 3 displays the changes in corneal wavefront aberrations following SMILE. Vertical coma and the root mean square values of HOAs were significantly increased in both corneal anterior surface and total cornea after SMILE surgery compared to those measured before the surgery (all *P* < 0.001). Besides, no significant differences were found in the values of spherical aberration, horizontal coma, vertical trefoil, and horizontal trefoil between pre- and post-SMILE surgery (*P*=0.055 ~ 0.432 > 0.05).

## 4. Discussion

Our study indicated that 100% eyes reached 20/20 or better in UDVA at 7-year follow-up. The efficacy and safety indices were 1.14 ± 0.19 and 1.19 ± 0.20, respectively. SMILE showed excellent safety and efficacy for moderate-to-high myopia correction. Han et al. [11] demonstrated that the UDVA was 20/20 or better in 92% of eyes at 4 years after SMILE, and the efficacy and safety indices were 1.07 ± 0.16 and 1.16 ± 0.14. Blum et al. [10] reported the data from 56 eyes after SMILE during a 5-year follow-up period. They found the efficacy and safety indices were 0.9 and 1.2, respectively. Agca et al. [14] suggested that efficacy and safety indices were 0.98 ± 0.21 and 1.04 ± 0.15 at 5 years after SMILE for mild and moderate myopia. These results were consistent with our study. Indeed, the long-term results (7 years) confirmed the efficacy and safety of the SMILE procedure.

In this study, 92% and 100% of eyes were within ±0.5 D and ±1.00 D of the targeted correction, respectively. During a 4-year follow-up period, Burazovitch et al. [12] found that 87% of eyes were within ±0.50 D of the intended correction in patients with high myopia. Moreover, in the study by Han et al. [11], 89% of eyes were within ±0.50 D of the intended correction and 100% were within ±1.00 D. Similarly, Li et al. [15] reported that 90% and 100% of eyes were within ±0.5 D and ±1.00 D of the intended correction, respectively, at 5 years after SMILE. Our findings were similar with the abovementioned studies. Besides, Agca et al. [13] demonstrated 5-year results of 37 cases with high myopia, in which 59% of them are within ±0.50 D of the intended correction, and 92% of them are within ±1.00 D. They clarified that it was most likely attributed to a greater tendency toward undercorrection. Agca et al. [14] showed that 93% of eyes were within ±0.50 D of the intended correction and 100% were within ±1.00 D among patients with mild-to-moderate myopia. The predictability after 7 years of SMILE in the current study was slightly better, which may be related to surgical parameters' design and calculation. Additionally, the use of a new nomogram could help improve the accuracy of refractive correction [17].

A mean refractive regression of −0.17 D was observed between 1 month and 7 years postoperatively, which corresponded to a regression rate of −0.02 D per year. Blum et al. [18] reported the 10-year refraction after SMILE was −0.35 ± 0.66 D, corresponding to a regression of −0.30 D only. Pedersen et al. [4] found a regression of −0.08 D from 3 months to 3 years after SMILE. These studies were similar with ours. However, Agca et al. [13] reported 5-year outcomes of patients with high myopia after SMILE and found a statistically significant relationship between 1- and 5-year postoperative regression. They concluded that regression could start in the early postoperative period. In other long-term studies [19, 20] of LASIK for moderate and high myopia, a mean regression of 0.63 to 0.97 D was observed after 6 or 7 years. Li et al. [15] compared the 5-year results between SMILE and FS-LASIK, and the average regression of the SMILE group and FS-LASIK group from 6 months to 5 years postoperatively was found to be −0.02 D and −0.12 D. SMILE maintained the stability of refraction better than FS-LASIK. This maybe contributed to better preservation of corneal biomechanics [21].

We also found the changes in MCBC and PCE before and 7 years after the surgery were not statistically significant. Our previous studies [22, 23] revealed that the posterior corneal surface remained stable 1 year and 3 years after SMILE. Similar results have been reported by Gyldenkerne et al. [24], suggesting that the structure of cornea remains stable after SIMLE. Wang et al. [25] evaluated PCE change and corneal biomechanical parameters such as corneal hysteresis (CH) and the corneal resistance factor (CRF) in eyes after SMILE and the FS-LASIK group and observed PCE change became greater in the FS-LASIK group than that in the SMILE group at 12 months after surgery. Meanwhile, FS-LASIK demonstrated a greater reduction of CRF after operation. However, further investigations with larger sample sizes and longer follow-up periods are needed.

In addition, the results of this study indicated that the values of vertical coma increased remarkably 7 years after SMILE, followed by HOA values. There were no significant changes in the values of spherical aberrations and trefoil. All the four wavefront aberrations remained relatively stable at the indicated postoperative time points. These results were consistent with the findings of Agca et al. [26]. Pedersen et al. [4] show the decrease in HOA and spherical aberration from 3 months to 3 years after SMILE, probably due to the long-term corneal remodeling after the surgery. A recent study by Gyldenkerne et al. [27] demonstrated that the spherical aberration did not change significantly at 3 months after SMILE surgery. Besides, Lee et al. [28] compared the corneal aberrations between SMILE and transepithelial photorefractive keratectomy (PRK) after 6 months and found smaller spherical aberration and larger coma values in the SMILE group.

The present study has several limitations. First, the sample size was relatively small. Second, patients with moderate and high myopia were categorized into a single group. Finally, this study focused on corneal aberrations instead of all eye aberrations.

In conclusion, this long-term follow-up study reveals that SMILE is an effective, safe, and stable surgical approach for myopia correction, with good predictability and relatively constant corneal stability and wavefront aberrations.

## Figures and Tables

**Table 1:** Baseline characteristics of patients.

Parameters	Mean ± SD (range)

Gender (*n*/*n*)	9 males/17 females
Age (years)	28.27 ± 7.76 (18 to 43)
Average follow-up (months)	82.53 ± 8.5 (73 to 101)
IOP (mmHg)	16.50 ± 1.69 (13.6 to 19.5)
Axial length	26.02 ± 0.80 (24.12 to 27.29)
MRSE (D)	−6.33 ± 1.19 (−8.75 to −4.38)
Manifest sphere (D)	−5.95 ± 1.14 (−8.50 to −4.25)
Manifest cylinder (D)	−0.76 ± 0.48 (−1.50 to 0)
Lenticule thickness (*μ*m)	125.92 ± 18.32 (95 to 164)

D = diopters; MRSE = manifest refraction spherical equivalent; IOP = intraocular pressure.

**Table 2:** Corneal stability analysis of Scheimpflug imaging.

Parameters	Pre	1 month	1 year	5 year	7 year	*P*

CCT	544.11 ± 28.19	435.06 ± 39.63^a^	439.89 ± 38.23^ab^	445.56 ± 36.10^ab^	442.06 ± 37.72^ab^	<0.001
MCFC	43.53 ± 0.27	38.56 ± 0.44^a^	38.87 ± 0.43^ab^	39.00 ± 0.40^ab^	39.15 ± 0.40^abc^	<0.001
MCBC	−6.37 ± 0.23	−6.38 ± 0.22	−6.34 ± 0.20	−6.36 ± 0.23	−6.34 ± 0.21	0.089
PCE	3.22 ± 2.76	1.50 ± 2.01^a^	0.67 ± 2.22^a^	1.94 ± 3.32	1.72 ± 2.54	<0.001
ΔPCE	—	−0.71 ± 3.14	−1.82 ± 4.61	−0.88 ± 4.06	−1.76 ± 4.02	0.463

CCT = central corneal thickness; MCFC = mean corneal front curvature (from central 4 mm diameter cornea); MCBC = mean corneal back curvature (from central 4 mm diameter cornea); PCE = posterior central elevation; ΔPCE = subtracting preoperative PCE from postoperative PCE; ^a^vs pre values of statistical significance (*P* < 0.05); ^b^vs 1-month values of statistical significance (*P* < 0.05); ^c^vs 1-year values of statistical significance (*P* < 0.05).

**Table 3:** Preoperative versus postoperative corneal wavefront aberrations for a 5 mm pupil.

Parameters	Pre	1 month	1 year	5 years	7 years	*P*

*Corneal (front)*						
SA	0.13 ± 0.05	0.16 ± 0.08	0.13 ± 0.16	0.15 ± 0.07	0.14 ± 0.05	0.414
Coma (vertical)	−0.03 ± 0.13	−0.31 ± 0.26^a^	−0.35 ± 0.22^a^	−0.31 ± 0.22^a^	−0.34 ± 0.21^a^	<0.001
Coma (horizontal)	−0.01 ± 0.04	0 ± 0.16	−0.02 ± 0.14	−0.03 ± 0.15	−0.08 ± 0.14	0.102
Trefoil (vertical)	0.01 ± 0.06	0.05 ± 0.06	0.04 ± 0.08	−0.01 ± 0.05	−0.01 ± 0.04	0.135
Trefoil (horizontal)	0.01 ± 0.03	0.05 ± 0.08	0.01 ± 0.06	0.01 ± 0.06	0.02 ± 0.07	0.282
RMS HOA	0.21 ± 0.02	0.45 ± 0.06^a^	0.45 ± 0.05^a^	0.43 ± 0.04^a^	0.43 ± 0.05^a^	<0.001

*Corneal (back)*						
SA	−0.08 ± 0.02	−0.08 ± 0.02	−0.08 ± 0.02	−0.08 ± 0.02	−0.09 ± 0.01^b^	0.001
Coma (vertical)	−0.02 ± 0.03	0 ± 0.03^a^	0 ± 0.03^a^	0 ± 0.03^a^	0 ± 0.03	<0.001
Coma (horizontal)	0.01 ± 0.02	0.01 ± 0.03	0.01 ± 0.03	0.01 ± 0.03	0.01 ± 0.03	0.244
Trefoil (vertical)	0.01 ± 0.02	0 ± 0.03	0 ± 0.02	−0.02 ± 0.03	−0.01 ± 0.04	0.066
Trefoil (horizontal)	0 ± 0.02	0.01 ± 0.02	0.02 ± 0.04	0.02 ± 0.03	0.01 ± 0.04	0.432
RMS HOA	0.10 ± 0.02	0.11 ± 0.02	0.11 ± 0.02	0.11 ± 0.02	0.12 ± 0.03	0.033

*Corneal (total)*						
SA	0.11 ± 0.05	0.14 ± 0.09	0.11 ± 0.07	0.13 ± 0.08	0.11 ± 0.05	0.359
Coma (vertical)	−0.04 ± 0.13	−0.35 ± 0.38^a^	−0.38 ± 0.23^a^	−0.34 ± 0.23^a^	−0.38 ± 0.22^a^	<0.001
Coma (horizontal)	0 ± 0.05	0.01 ± 0.17	−0.01 ± 0.14	−0.03 ± 0.14	−0.09 ± 0.14	0.055
Trefoil (vertical)	0.02 ± 0.07	0.05 ± 0.07	0.05 ± 0.10	−0.03 ± 0.07^b^	−0.02 ± 0.05^b^	<0.001
Trefoil (horizontal)	0.01 ± 0.04	0.07 ± 0.09	0.03 ± 0.07	0.04 ± 0.07	0.03 ± 0.07	0.247
RMS HOA	0.21 ± 0.07	0.49 ± 0.25^a^	0.48 ± 0.21^a^	0.46 ± 0.18^a^	0.46 ± 0.21^a^	<0.001

HOAs = higher-order aberrations; SA = spherical aberration; ^a^vs. pre values of statistical significance (*P* < 0.05); ^b^vs. 1-month values of statistical significance (*P* < 0.05); ^c^vs. 1-year values of statistical significance (*P* < 0.05). Data are presented as mean ± SD.

## Data Availability

Data analyzed during the current study are available from the corresponding author upon reasonable request.
